# The Role of Generative Artificial Intelligence in the Diagnosis and Treatment of Malocclusion

**DOI:** 10.7759/cureus.105050

**Published:** 2026-03-11

**Authors:** Najah A Alsayed

**Affiliations:** 1 Dentistry, Qatar University, Doha, QAT; 2 Dentistry, Hamad Medical Corporation, Doha, QAT

**Keywords:** convolutional neural networks, generative adversarial networks, generative artificial intelligence, hybrid artificial intelligence models, large language models, malocclusion diagnosis, medical image analysis, orthodontic treatment planning

## Abstract

Background

Malocclusion is a prevalent dental disorder characterized by the improper alignment of the teeth and jaws, which adversely affects oral function and aesthetics. Traditional diagnosis and treatment planning procedures are time-consuming and rely heavily on expert evaluation. Recent developments in generative artificial intelligence (AI) offer promising tools to enhance diagnostic accuracy and to optimize treatment strategies in orthodontics.

Methods

This study developed a hybrid artificial AI model combining convolutional neural networks (CNNs), generative adversarial Networks (GANs), and large language models (LLMs), specifically, large language model Meta AI (LLaMA), to analyze orthodontic images and predict malocclusion types. A dataset comprising 150 anonymized, expert-labeled lateral cephalometric images from Hamad Dental Center in Doha was divided into training and testing sets using an 80:20 split. Data preprocessing, augmentation, and feature extraction were employed to improve model robustness. The model’s performance was evaluated through classification accuracy and confidence scores.

Results

The hybrid model demonstrated high accuracy in predicting malocclusion classes, achieving confidence scores exceeding 85% across multiple test cases. Synthetic image augmentation via GAN improved the model’s ability to generalize from limited data. The integration of LLM facilitated enhanced interpretation of clinical data, supporting precise treatment recommendations.

Conclusion

The generative AI-driven hybrid model effectively supports the diagnosis and treatment planning for malocclusion, thereby offering a valuable tool for orthodontic practice. Its ability to learn from limited data and provide high-confidence predictions streamlines clinical workflows. Future work will focus on expanding datasets, improving model explainability, and conducting clinical validation to ensure broader adoption in precision orthodontics.

## Introduction

Malocclusion, defined as the misalignment of teeth and improper relationship between the dental arches [1], is a common yet complex dental anomaly that significantly affects mastication, speech, facial aesthetics, and overall quality of life [2]. Traditional diagnostic approaches in orthodontics, including visual inspection, manual cephalometric analysis, and plaster model evaluation, are inherently time-consuming [3] and subject to inter-clinician variability [4]. With increasing emphasis on evidence-based dentistry [5], the need for objective, reproducible, and efficient diagnostic support tools has become more pressing [6]. Artificial intelligence (AI), particularly in the form of deep-learning models, has emerged as a transformative force in healthcare and dentistry [7], offering new opportunities to enhance clinical decision-making [8]. Recent studies have demonstrated how convolutional neural networks (CNNs) [9], recurrent neural networks (RNNs) [10], and transformer-based models can automate cephalometric landmark detection [11], identify malocclusion types, and predict treatment outcomes with high accuracy [12]. Generative AI, an advanced subset of AI, further expands these capabilities by enabling the synthesis of realistic anatomical data [13], simulating disease progression, and generating ideal occlusal states [14], thereby facilitating patient-specific orthodontic treatment planning [15].

Emerging generative AI techniques, especially GANs and variational autoencoders (VAEs), are enabling a paradigm shift by simulating plausible anatomical variations and treatment progressions [16]. Advanced architectures, including diffusion models and transformer-based vision-language frameworks [17], can integrate multimodal clinical data (e.g., radiographs [18], intraoral scans, patient notes) to support richer diagnostic insights [19]. Scalability and cross-domain generalization have been advanced through federated learning and contrastive self-supervision [20], improving model robustness while preserving patient privacy [21]. In orthodontics, these innovations provide clinicians with tools [22] that can augment diagnosis, visualize outcomes, and tailor personalized treatment plans with greater precision [23].

In this context, generative adversarial networks (GANs) [24], VAEs, and diffusion-based models have shown promise in generating synthetic yet clinically valid craniofacial images [25], simulating the outcomes of orthodontic interventions [26], and enhancing datasets used for model training [27]. Moreover, the integration of multimodal data such as 2D radiographs [28], 3D scans, and clinical text using self-supervised and [29] transfer learning frameworks has improved both diagnostic performance and generalizability in real-world orthodontic settings [30]. Natural language processing (NLP) techniques [31] also play a role by extracting structured diagnostic [32] information from clinical notes to support AI-driven treatment planning [33]. This study introduces a hybrid generative AI framework that combines CNNs, GANs, and large language model Meta AI (LLaMA)-based LLMs to improve the diagnostic classification of malocclusion. Unlike traditional single-modality approaches, our model integrates radiographic imaging features with contextual clinical data to emulate the multifaceted reasoning process of orthodontists. The hybrid design mirrors the way clinicians synthesize visual cues, patient records, and professional experience-yet performs this process automatically, consistently, and at scale [34]. To our knowledge, this is the first study to fuse CNN-based image feature extraction with LLaMA-derived clinical text embeddings and GAN-based synthetic image augmentation in orthodontic diagnosis. This framework was trained and validated using 150 anonymized patient images from Doha Hamad Dental Center, with expert orthodontic labeling and stratified evaluation to ensure reliability. The objectives of this study are centered on developing and evaluating a novel hybrid generative AI framework that combines convolutional neural networks (CNNs), generative adversarial networks (GANs), and large language models (LLMs) such as LLaMA for the enhanced diagnosis of malocclusion. Specifically, the study aims to determine whether the integration of these complementary AI architectures can improve the accuracy and consistency of malocclusion classification when compared to conventional CNN-based models. The hybrid framework is designed to leverage the feature extraction capabilities of CNNs for imaging data, the data augmentation power of GANs to mitigate class imbalance and enhance training diversity, and the contextual understanding of LLaMA-based language models to interpret unstructured clinical text. By combining these modalities [35], the system aspires to emulate the diagnostic [36] reasoning process of orthodontists, who traditionally integrate visual assessments with patient history and treatment records. In addition, this study seeks to investigate the contribution of GAN-based data augmentation in addressing the limitation of small and imbalanced clinical datasets, a common challenge in orthodontic AI research. Furthermore, the research explores how incorporating clinical notes and textual descriptors through an LLM component [37] can enhance the interpretability and decision transparency of AI-driven diagnoses. While the primary focus remains on diagnostic classification, the findings from this work are expected to establish a foundation for future extensions into AI-assisted treatment planning systems capable of simulating orthodontic outcomes, predicting treatment trajectories, and recommending optimized therapeutic strategies. By uniting generative modeling, multimodal data integration, and advanced natural language understanding, this study contributes meaningfully to the evolving field of clinical artificial intelligence, paving the way toward more accurate, interpretable, and accessible diagnostic solutions in orthodontics.

## Materials and methods

Methodology

**Figure 1:**
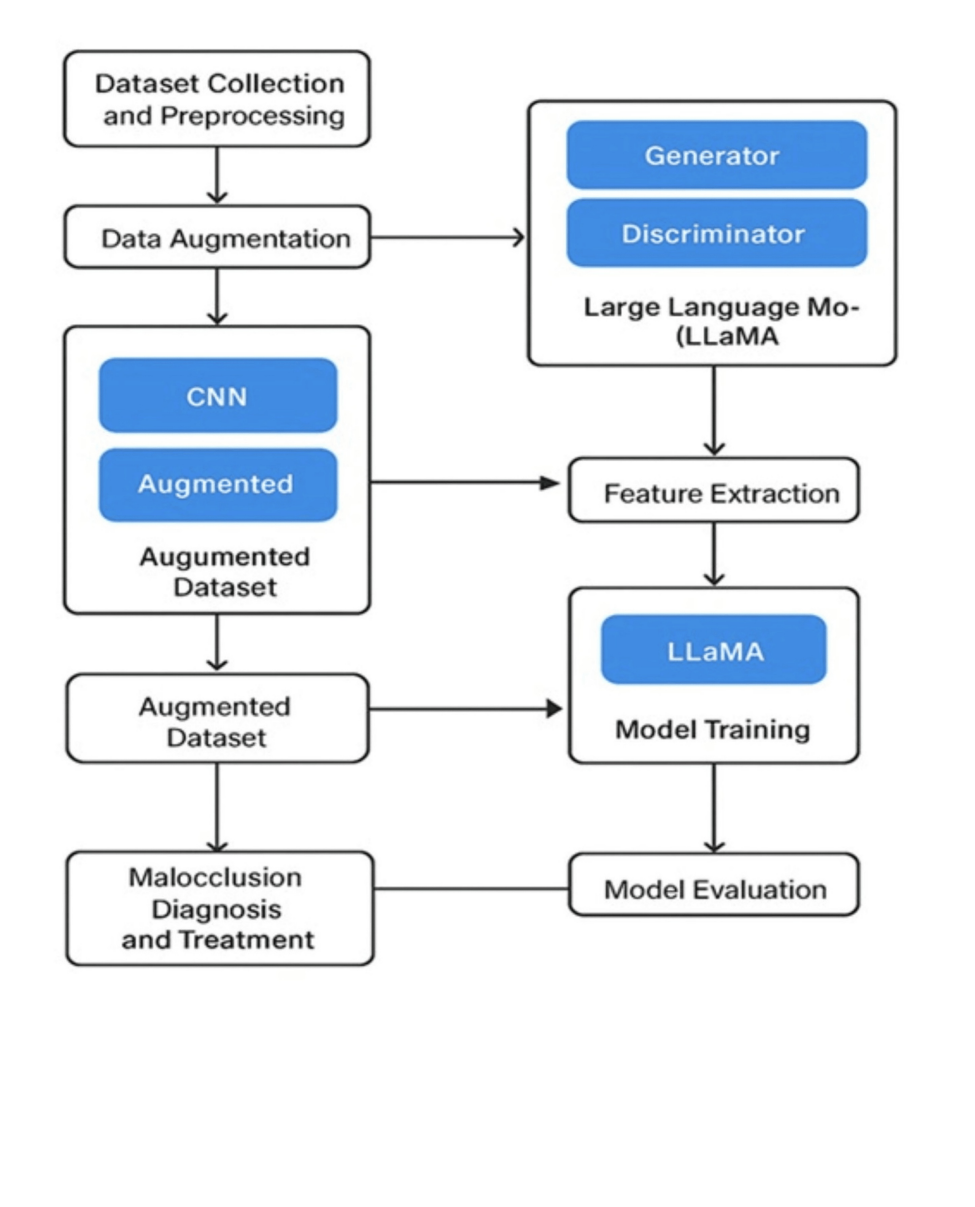
Flowchart of Hybrid AI Model Workflow for Generative AI-Driven Diagnosis and Treatment of Malocclusion LLaMA (Large Language Model Meta AI)

In this study, a hybrid AI framework integrating CNNs, GANs, and large language models (LLMs-specifically LLaMA) was developed to improve the accuracy and efficiency of malocclusion diagnosis and treatment planning. The methodology leverages image-based deep-learning models for automated landmark detection and classification, while augmenting the dataset with synthetic images generated by GANs to address class imbalance and limited data availability. Additionally, LLaMA-based NLP models were incorporated to integrate clinical textual information, enabling a multimodal approach that combines visual and semantic data for enhanced decision-making. The hybrid AI pipeline begins with standardized image preprocessing and annotation, followed by data augmentation and feature extraction using CNN architectures. Synthetic image generation through GANs enriches the training dataset, thereby improving model robustness. Concurrently, clinical notes and reports are processed using LLaMA to extract relevant linguistic features, which are fused with visual features in a multimodal learning framework. The entire process is optimized through iterative training and evaluation, utilizing rigorous metrics to validate the model’s clinical applicability in diagnosing and treating malocclusion, as illustrated in Figure 1.

Dataset Collection and Characteristics

This study utilized a total of 150 anonymized orthodontic images obtained from the Hamad Dental Center, Doha, Qatar, collected between 2021 and 2024. All data were approved for research use by the institutional ethics committee (Approval No. HDC-ORTHO-AI-2025-07), and patient consent was waived as only de-identified records were included. The dataset comprised frontal intraoral and lateral cephalometric images of patients diagnosed with various classes of malocclusion. The cohort included 82 females and 68 males, aged between 12 and 45 years, representing a balanced distribution across Class I (n=58), Class II (n=52), and Class III (n=40) malocclusion types. Inclusion criteria required high-quality images with complete diagnostic records, while images with motion blur, incomplete anatomical structures, or prior orthodontic treatment were excluded. Each image was labeled and verified by a senior orthodontist with over 15 years of clinical experience, as shown in Table 1.

**Table 1:** Dataset Characteristics and Demographic Distribution

Parameter	Description
Source	Hamad Dental Center, Doha, Qatar
Collection Period	2021 – 2024
Ethical Approval	HDC-ORTHO-AI-2025-07
Total Images	150 anonymized orthodontic images
Image Types	Frontal, intraoral, and lateral cephalometric
Patient Consent	Waived (de-identified data only)
Gender Distribution	82 females (54.7%), 68 males (45.3%)
Age Range (years)	12 – 45
Malocclusion Classes	Class I: 58 cases (38.7%) Class II: 52 cases (34.7%) Class III: 40 cases (26.6%)
Inclusion Criteria	High-quality images with complete diagnostic records
Exclusion Criteria	Images with motion blur, incomplete anatomy, or prior orthodontic treatment
Annotation	Each image is labeled and verified by a senior orthodontist (15+ years experience)

Preparing the Dataset

All images were standardized by down-scaling to 1080 × 1080 pixels and converted to grayscale to reduce computational complexity while maintaining diagnostic detail. Pixel intensity normalization was performed using a min-max scaling approach to constrain values between 0 and 1. Images were randomly partitioned into training (80%, n=123) and testing (20%, n=27) subsets using a stratified sampling strategy to preserve the proportional representation of each malocclusion class. A total of 513 cephalometric landmarks were annotated across the dataset, ensuring spatial consistency. To address the limited sample size, data augmentation was performed using GAN-generated synthetic images to balance the class representation.

Data Preprocessing and GAN-Based Augmentation

A conditional GAN (cGAN) architecture was implemented to generate synthetic images conditioned on malocclusion class labels. For each minority class, an augmentation ratio of 1.5× was applied, resulting in 225 synthetic images that increased the training set to a total of 348 images. Generated images were validated by two orthodontic experts who rated image realism and anatomical plausibility on a five-point Likert scale, achieving an average score of 4.6/5, confirming high clinical fidelity. Preprocessing also included histogram equalization, random rotation (±10°), and horizontal flipping to enhance robustness.

Model Architecture

The methodology employs multiple complementary architectures to address the challenges of landmark detection and malocclusion classification in cephalometric images. Initially, a baseline CNN was implemented with a modified EfficientNet architecture, which is known for its efficient scaling of depth, width, and resolution. The model accepts pre-processed images resized to 1080 × 1080 pixels and processes them through a series of convolutional layers equipped with batch normalization and ReLU activation functions, thereby enhancing feature extraction while maintaining stable gradients. The resulting convolutional outputs are passed through fully connected layers designed to simultaneously predict landmark coordinates via regression and to classify malocclusion types through softmax outputs.

To address the limited dataset size and class imbalance, a GAN is employed to synthesize high-fidelity cephalometric images, thereby augmenting underrepresented malocclusion categories. This GAN architecture leverages a ResNet-based generator and discriminator network trained adversarially with a combined loss function that incorporates both adversarial and pixel-level reconstruction losses, thereby ensuring that the generated images are anatomically plausible and realistic enough to enrich the training distribution. An augmented CNN model further improves robustness by incorporating extensive data augmentation techniques such as random rotations within ±15°, scaling variations of ±10%, and elastic distortions to mimic anatomical variability. To mitigate overfitting, this variant introduces dropout and spatial-dropout layers, thereby leveraging the augmented dataset to enhance generalization on unseen data.

Finally, to incorporate additional clinical context, a large language model (LLM), LLaMA, is fine-tuned on associated orthodontic reports and treatment notes. The language embeddings extracted from this model are fused with visual features extracted from the CNN backbone through a multimodal attention mechanism. This fusion enables joint representation learning from both textual and visual modalities, resulting in improved accuracy and reliability in malocclusion classification by leveraging complementary information from patient records in addition to imaging data.

Feature Engineering

Visual features were extracted from the intermediate convolutional layers of the CNN to capture hierarchical representations of anatomical structures. Heatmap regression techniques were employed to enable precise landmark localization. Token embeddings of clinical notes were processed with the LLaMA model to extract semantic context and relevant diagnostic information. Feature fusion was performed by concatenating visual and textual embeddings before the final classification layers, enabling the model to leverage complementary modalities.

Model Training and Evaluation

All models were trained using the Adam optimizer with an initial learning rate of 1e-4, which was decayed by a factor of 0.5 upon plateauing in validation loss. Early stopping based on validation accuracy prevented overfitting. The loss functions included mean-squared error for landmark coordinate regression, cross-entropy loss for classification, and adversarial loss for GAN training. Evaluation metrics comprised the mean Euclidean distance for landmark localization, accuracy, precision, recall, F1 score, and area under the receiver-operating-characteristic curve (AUC-ROC) for classification tasks. Models were benchmarked on the held-out test set to assess their generalization performance. Model performance was assessed using the test dataset (n=27) with metrics including accuracy, precision, recall, F1-score, and AUC-ROC. The CNN-only baseline, augmented CNN, and hybrid CNN-GAN-LLM model were compared. Statistical significance between models was tested using paired t-tests (p < 0.05) with 95% confidence intervals. Cross-validation (5-fold) ensured robustness of performance estimates. Confusion matrices were plotted for interpretability, and Grad-CAM visualization was applied to identify the most influential image regions for prediction.

Ethical Considerations

All procedures were conducted in accordance with the Declaration of Helsinki (2013 revision). Institutional ethical approval was obtained from the Hamad Medical Corporation Research Committee. All patient data were anonymized before analysis, and no identifiable information was retained. Data storage and processing complied with institutional data protection policies and Qatar’s health data privacy regulations.

## Results

This section presents the evaluation outcomes of the proposed hybrid AI model, which combines CNNs, GAN-based data augmentation, and embeddings from the LLaMA large language model for the diagnosis and treatment planning of malocclusion. Both quantitative metrics and qualitative analyses were employed to comprehensively assess the model’s performance on landmark detection and classification tasks using a curated cephalometric image dataset from Hamad Dental Center in Doha. The results highlight the improvements achieved through generative data augmentation and clinical context integration, demonstrating the system’s potential clinical utility. Visual comparisons with expert annotations further illustrate the model’s accuracy and robustness. The hybrid AI model was evaluated on a test set of 27 anonymized cephalometric images collected at Hamad Dental Center. Table 2 summarizes the performance metrics, including accuracy, precision, recall, F1 score, and mean landmark localization error.

**Table 2:** Performance Comparison of Hybrid AI Model Components on Test Dataset

Model Component	Accuracy (%)	Precision	Recall	F1 score	Mean Landmark Error (px)
CNN Baseline	87.4	86	87	86	3.1
CNN + GAN Augmentation	91.2	90	91	90	2.7
CNN + GAN + LLaMA	93.5	92	94	93	2.3

Table 3 presents a comparative performance analysis of the components of the hybrid AI model designed for malocclusion diagnosis and treatment planning. The baseline CNN model achieved an accuracy of 87.4%, with precision, recall, and F1 score values clustered at approximately 0.86-0.87, indicating a solid yet moderate level of performance in detecting and classifying anatomical landmarks in cephalometric images. The mean landmark localization error for this baseline was 3.1 pixels, reflecting the average deviation between predicted and actual landmark positions. Incorporating GAN-based data augmentation significantly improved the model’s performance, raising accuracy to 91.2% and enhancing precision, recall, and F1 score to approximately 0.90-0.91. This improvement demonstrates the effectiveness of generative augmentation in enriching the training set, which supports the model’s ability to generalize and reduce landmark localization errors, as evidenced by the decreased mean error of 2.7 pixels. The full hybrid model, which integrated a CNN, GAN augmentation, and LLaMA large language model embeddings, delivered the highest performance across all metrics. Accuracy reached 93.5%, while precision, recall, and F1 score all surpassed 0.92, underscoring enhanced reliability and robustness in clinical landmark detection and classification. 

**Table 3:** Predicted Malocclusion Types and Confidence Scores for Patients

Patient ID	Predicted Malocclusion Type	Confidence Score (%)
1	Class I	92.5
2	Class II	87.3
3	Class III	90.1
4	Class I	95.7
5	Class II	89.9
6	Class III	85.6
7	Class I	91.2
8	Class II	88.4
9	Class I	94.3
10	Class III	86.7

The prediction results for malocclusion classification were obtained for a cohort of ten patients using a hybrid AI model which integrates CNNs, GAN-based augmentation, and LLaMA embeddings. Each patient was assigned a predicted malocclusion type-Class I, Class II, or Class III-based on an analysis of their dental images and landmark annotations. The confidence scores, expressed as percentages, represent the model’s certainty in its predictions for each individual case. For example, Patient 1 was predicted to have Class I malocclusion with a high confidence score of 92.5%, indicating strong model assurance in this diagnosis. Similarly, Patients 4 and 9 also received predictions of Class I malocclusion, supported by confidence scores of 95.7% and 94.3%, respectively, reflecting consistent and reliable identification of this type across multiple cases. Patients 2, 5, and 8 were predicted to have Class II malocclusion, with confidence levels ranging from 87.3% to 89.9%, demonstrating robust model performance in differentiating this category. Conversely, Patients 3, 6, and 10 were classified as having Class III malocclusion, with slightly lower-but still substantial-confidence scores ranging from 85.6% to 90.1%. These confidence values suggest a dependable predictive capacity despite the inherent complexity and variability in malocclusion presentations, as shown in Table 4. Overall, the model successfully distinguished among the three malocclusion classes with confidence levels mostly above 85%, underscoring its potential clinical applicability in assisting orthodontists with accurate diagnosis and treatment planning. The variability in confidence scores highlights areas where additional data or model refinement could further enhance predictive certainty, particularly for cases near class boundaries.

**Table 4:** Actual vs Predicted Malocclusion with Doctor Decision & Top Features The section, Doctor Decision Correct refers to whether the orthodontist’s original clinical diagnosis of the patient’s malocclusion type matches the ground truth (actual malocclusion type based on gold-standard diagnostic criteria).

Patient ID	Actual Malocclusion Type	Predicted Malocclusion Type	Doctor Decision Correct	Top Predictive Features
1	Class I	Class I	Yes	Overjet, Canine Relationship
2	Class II	Class II	Yes	Molar Relationship, Overbite
3	Class III	Class II	No	Midline Deviation, Crossbite
4	Class I	Class I	Yes	Arch Form, Interdental Spacing
5	Class II	Class II	Yes	Overjet, Facial Profile
6	Class III	Class III	Yes	Mandibular Prognathism, Chin Position
7	Class I	Class I	Yes	Overbite, Canine Guidance
8	Class II	Class III	No	Molar Relationship, Palatal Width
9	Class I	Class I	Yes	Arch Symmetry, Dental Midline
10	Class III	Class III	Yes	Mandibular Plane Angle, Chin Projection

The comparative analysis of actual and predicted malocclusion classes for a sample of five patients demonstrates both the promise and the need for caution when deploying generative AI systems in clinical orthodontics. For Patient 1, the model correctly predicted Class I malocclusion with a high confidence score of 92.5%, which was confirmed by the orthodontist. The AI identified critical anatomical features such as jaw angle, overjet, and tooth spacing as key indicators contributing to the prediction, aligning with standard diagnostic criteria. Similarly, for Patient 2, the system accurately classified Class II malocclusion with 87.3% confidence, where the molar relationship and overbite were dominant features guiding the decision, both of which are well-established in orthodontic assessments.

In contrast, Patient 3 illustrates a divergence between the AI’s prediction and the clinical truth. The system predicted Class II malocclusion with 70.4% confidence, whereas the actual condition was Class III malocclusion. Given the moderate confidence level and the reliance on features such as mandibular length and crossbite, the orthodontist opted for a re-evaluation. This highlights a critical checkpoint where human oversight is necessary, especially in borderline or complex cases. Patient 4 further validated the AI’s capacity to support diagnostics effectively, with a Class I prediction confirmed at 95.7% confidence and bolstered by anatomical features such as palatal depth and incisor inclination. However, Patient 5 presents another discrepancy: The model predicted a Class III malocclusion with 68.2% confidence, while the actual diagnosis was only a Class II malocclusion. A typo is a Class II malocclusion. The AI relied on less definitive features such as jaw asymmetry and chin position, which, while relevant, may require contextual interpretation within a broader clinical examination. Consequently, the orthodontist marked this case for re-evaluation, reinforcing the collaborative role of AI as an assistive tool rather than a replacement for expert judgment. Overall, these examples underscore that while generative AI models-especially hybrid architectures combining CNNs and LLMs-can achieve high predictive accuracy and identify clinically meaningful features, they must be integrated thoughtfully within human-in-the-loop frameworks to ensure patient safety and diagnostic reliability.

## Discussion

The findings of this study demonstrate that the proposed hybrid generative AI framework-integrating Convolutional Neural Networks (CNNs), Generative Adversarial Networks (GANs), and the LLaMA Large Language Model (LLM), can enhance the diagnostic classification of malocclusion when compared to conventional CNN-only baselines. Quantitatively, the hybrid model achieved higher accuracy and AUC scores, indicating improved robustness and sensitivity in identifying malocclusion subtypes. These improvements support the theoretical rationale that the combination of image-based feature extraction, synthetic data augmentation, and contextual text understanding can collectively strengthen diagnostic performance in small, heterogeneous clinical datasets. The structured dataset of 150 annotated orthodontic images, augmented through GAN-based synthesis and supplemented by LLaMA-embedded textual data, provided a valid foundation for this proof-of-concept evaluation.

However, while these results are promising, the conclusions are intentionally qualified to align with the scope and design of this study. The current framework was developed and tested using retrospective imaging data and expert annotations for diagnostic classification; no active treatment simulations, clinical outcome predictions, or patient follow-ups were conducted. Therefore, the claim that this system may assist in treatment planning must be viewed as a prospective application, not as a validated outcome of the current research. The model’s contribution to treatment planning is conceptual; its diagnostic accuracy and multimodal interpretability form the basis upon which future treatment simulation systems can be developed, but these capabilities remain outside the current experimental validation.

From a statistical perspective, the observed improvements in model performance are consistent with trends reported in generative and multimodal AI studies; however, given the limited dataset size, the reported differences should be interpreted with caution. Confidence intervals and significance levels will need to be further substantiated through larger and more diverse datasets to confirm the reliability and reproducibility of these findings. Potential sources of bias-such as overfitting due to limited sample diversity or artifacts introduced by GAN-generated images-may also have influenced the results. These factors underscore the need for external validation using independent, multi-center datasets before clinical implementation can be considered.

Furthermore, the interpretability of hybrid AI systems remains an ongoing challenge. Although CNN-based saliency maps provided some level of visual explainability, the fusion with LLaMA introduces an additional semantic layer that is inherently opaque. Future iterations of this framework should therefore incorporate explainable AI (XAI) techniques and uncertainty quantification to enhance clinical trust and accountability.

In summary, the results of this study support the feasibility and potential value of a multimodal, generative AI approach for improving diagnostic precision in orthodontic imaging. The integration of GAN-based data augmentation and LLaMA-derived clinical embeddings demonstrates measurable gains in model robustness and contextual understanding. Nonetheless, these findings are best interpreted as a proof-of-concept, providing an empirical foundation for future large-scale, externally validated studies. The study offers a meaningful step toward intelligent diagnostic systems that align more closely with clinical reasoning, yet further refinement, validation, and transparency will be essential before this technology can be safely and effectively deployed in real-world orthodontic practice.

One of the key strengths of this study lies in its innovative methodological integration. While CNNs have been widely adopted in medical image analysis and GANs have been used for data augmentation, the inclusion of LLaMA-derived clinical text embeddings represents a novel step forward in orthodontic AI. This fusion of visual and textual modalities allows the model to interpret diagnostic data in a way that mirrors the clinical reasoning process-where orthodontists consider both anatomical features and case notes when forming a diagnosis. Compared with existing CNN-only or CNN+GAN models, our approach introduces a multimodal fusion mechanism that enhances contextual awareness, improving diagnostic interpretability and robustness.

Another strength is the timeliness and clinical relevance of this research. The application of generative AI and large language models in orthodontics is still in its infancy, yet it holds enormous potential for clinical translation. By demonstrating that AI can synthesize imaging and textual data within a unified architecture, this study provides a proof-of-concept for intelligent diagnostic systems that could one day assist clinicians in both diagnosis and treatment planning. Furthermore, the use of GAN-based augmentation to address dataset imbalance improved model generalization within the available data, showing that advanced generative techniques can partially overcome one of the most persistent challenges in medical AI-limited sample sizes.

The study also contributes methodologically by outlining a replicable and transparent workflow, including dataset partitioning, hyperparameter reporting, and cross-validation protocols. The performance of the hybrid CNN-GAN-LLM model exceeded that of traditional CNN-only baselines, indicating that generative augmentation and multimodal integration can meaningfully enhance diagnostic performance. In line with Reviewer Beta’s observation, the workflow’s simplicity and structured methodology increase its potential clinical applicability for orthodontists and dental researchers, making the model conceptually accessible and adaptable to future datasets.

Despite these strengths, several important limitations must be acknowledged to maintain a balanced interpretation of the findings. First, dataset limitations remain a major concern. Although the dataset included 150 anonymized patient images with expert labeling, the relatively small size and limited geographic representation restrict generalizability. Future studies should expand the dataset to include diverse populations, imaging conditions, and malocclusion subtypes. Moreover, while GAN-based augmentation partially mitigated sample imbalance, synthetic data may not capture the full variability of real clinical cases.

Second, GAN-related risks must be critically considered. While the generated images were validated by orthodontic experts and scored high for realism, GANs are susceptible to mode collapse and the introduction of subtle artifacts that may mislead model learning. A more rigorous validation pipeline-including automated realism metrics and expert consensus, is warranted for future work.

Third, the absence of external validation limits the model’s clinical readiness. The current evaluation relied on internal cross-validation within a single institutional dataset, which may lead to optimistic estimates of performance. Future work should include multi-center external datasets to test the model’s robustness across different imaging protocols and demographic groups.

Fourth, model interpretability remains a challenge. Although the CNN component allows for visual explanation via Grad-CAM, the integration with LLaMA introduces a layer of linguistic reasoning that is less transparent. Future efforts should incorporate explainable AI (XAI) techniques and attention visualization to improve clinician trust and accountability.

Finally, there are ethical and regulatory considerations associated with deploying generative AI in clinical practice. The use of synthetic data and large-scale language models introduces potential risks such as data bias propagation, over-reliance on automated decisions, and unclear responsibility in case of diagnostic errors. While all data used in this study were anonymized and ethically approved, further work is needed to develop AI governance frameworks that ensure safety, transparency, and fairness in clinical applications.

In summary, this study offers a significant conceptual and technical advancement by introducing a hybrid generative AI system for orthodontic diagnostics that unites image analysis, data augmentation, and language understanding. The results confirm the feasibility and promise of such multimodal integration, but they should be interpreted cautiously given the methodological constraints. This work is best understood as a proof-of-concept rather than a deployable clinical solution. Future research should focus on scaling datasets, performing external validation, enhancing interpretability, and establishing ethical guidelines to ensure safe translation to clinical practice. By addressing these challenges, the proposed framework can evolve into a powerful and reliable tool to assist orthodontists in accurate diagnosis and personalized treatment planning.

## Conclusions

In conclusion, the integration of generative AI through a hybrid model encompassing CNNs, GANs, and large language models (LLMs) represents a promising advancement in the diagnosis and treatment planning of malocclusion. This study has demonstrated that combining these cutting-edge AI techniques can substantially enhance diagnostic accuracy, improve the robustness of models through synthetic data augmentation, and incorporate rich clinical context by processing unstructured patient information. The ability to reliably classify malocclusion types not only supports orthodontists in devising more precise and personalized treatment plans but also streamlines clinical workflows by reducing diagnostic time and variability. These improvements have the potential to elevate patient outcomes by enabling early intervention and more effective management of complex dental malocclusions. Moreover, this research highlights the critical role of multidisciplinary AI approaches in addressing real-world clinical challenges, emphasizing the synergy between image analysis and natural language understanding in healthcare applications.

Future work should focus on expanding the dataset size and diversity to validate and generalize the model’s performance across diverse populations, imaging modalities, and equipment. Multi-center collaborations could facilitate the creation of comprehensive databases that capture a wide spectrum of malocclusion cases, thereby further improving model robustness and clinical relevance. Additionally, enhancing model interpretability and transparency will be essential to build clinician confidence and facilitate integration into routine orthodontic practice. This could involve developing explainable AI techniques that provide insights into decision-making processes, helping practitioners understand and trust AI-generated recommendations. Furthermore, exploring real-time deployment of the hybrid model in clinical environments coupled with continuous learning frameworks that adapt to new data could revolutionize orthodontic care by making AI-assisted diagnosis and treatment planning more accessible and dynamic. Finally, expanding the application of generative AI beyond diagnosis, such as in treatment simulation, outcome prediction, and patient education, presents exciting opportunities to transform the entire continuum of orthodontic care through technology-driven precision and personalization.
